# Kidney biopsy analysis of 14 cases of renal disease after unrelated cord blood transplantation

**DOI:** 10.1186/s12882-026-05034-5

**Published:** 2026-05-16

**Authors:** Masato Mizuta, Naoki Sawa, Tatsuya Suwabe, Yuki Oba, Akinari Sekine, Masayuki Yamanouchi, Hiroki Mizuno, Noriko Inoue, Kiho Tanaka, Eiko Hasegawa, Atsushi Wake, Junichi Hoshino, Kei Kono, Kenichi Ohashi, Yutaka Yamaguchi, Makoto Fukuda, Motoaki Miyazono, Takehiko Wada, Yoshifumi Ubara

**Affiliations:** 1 Nephrology Center and Okinaka Memorial Institute for Medical Research, Toranomon Hospital, Tokyo, Japan; 2 Department of Hematology, Toranomon Hospital, Tokyo, Japan; 3 Department of Nephrology, Tokyo Women’s Medical, University, Tokyo, Japan; 4 Department of Pathology, Toranomon Hospital, Tokyo, Japan; 5 Department of Human Pathology, Institute of Science Tokyo, Tokyo, Japan; 6 Yamaguchi’s Pathology Laboratory, Chiba, Japan; 7 Department of Nephrology, Saga University Internal Medicine, Saga, Japan

**Keywords:** Hematopoietic stem cell transplantation (HSCT), Unrelated cord blood transplantation (UCBT), Peripheral blood stem cell transplantation (PBSCT), Peripheral blood hematopoietic cell transplantation (PBHCT), Bone marrow transplantation (BMT), Chronic kidney disease (CKD)

## Abstract

**Objective:**

To evaluate kidney biopsy findings to clarify renal disease in patients after hematopoietic stem cell transplantation (HSCT) using unrelated umbilical cord blood transplantation (UCBT).

**Methods:**

We retrospectively examined 14 patients who underwent UCBT at Toranomon Hospital, Tokyo, Japan, from 2015 to 2023 and subsequently developed kidney injury requiring biopsy.

**Results:**

At biopsy, median urinary protein was 0.57 g/day (IQR, 0.27–1.67), median serum creatinine was 1.97 mg/dL (IQR, 1.81–2.6), and median eGFR was 25.3 mL/min/1.73 m^2^ (IQR, 18.1–34.2). In 13 of 14 patients, mesangiolysis, glomerular basement membrane (GBM) duplication, and subendothelial widening without thrombi were observed—lesions defined as glomerular microangiopathy (GMA). Immunofluorescence and electron microscopy revealed no immune deposits typical of membranous nephropathy. Eleven patients showed distinctive arterial and arteriolar changes termed vascular microangiopathy. Nine exhibited severe interstitial fibrosis and tubular basement membrane duplication involving > 50% of the cortex. Human leukocyte antigen (HLA) incompatibility was found in 13 patients (92.9%) and ABO incompatibility in nine (64.2%). C4d positivity in glomeruli or peritubular capillaries was detected in 12 patients (85.7%).

**Conclusion:**

The coexistence of glomerular, vascular, and tubulointerstitial microangiopathic lesions was associated with mild proteinuria and renal dysfunction after UCBT. These findings suggest a chronic endothelial injury process distinct from classical thrombotic microangiopathy.

## Introduction

Hematopoietic stem cell transplantation (HSCT) is a breakthrough treatment for refractory diseases, including hematopoietic malignancies and autoimmune diseases, classified into three types by stem cell source [1]: bone marrow transplantation (BMT) with cells from a relative or bone marrow bank (allogeneic [allo]) [2]; peripheral blood cells from the patient (autologous [auto]) or a relative (allo) collected before HSCT, referred to as peripheral blood stem cell transplantation (PBSCT; also called peripheral blood hematopoietic cell transplantation [PBHCT]); and [3] unrelated cord blood transplantation (UCBT) with cells from a cord blood bank.

HSCT is a toxic treatment involving high-dose chemotherapy, sometimes total-body radiotherapy, and long-term infectious disease treatments. Patients risk serious adverse reactions, including kidney disease [1]. Two types of kidney disease may develop years after HSCT: chronic kidney disease (CKD) with mild proteinuria but reduced renal function and nephrotic syndrome (NS) with severe proteinuria. Literature on kidney disease after HSCT is limited and heterogeneous [2–4].

We examined the clinical characteristics and renal pathology of 14 patients who developed renal disease after undergoing UCBT at our hospital. To our knowledge, this is the largest study to date of renal disease after UCBT.

## Materials and methods

### Study population

**Fig. 1:**
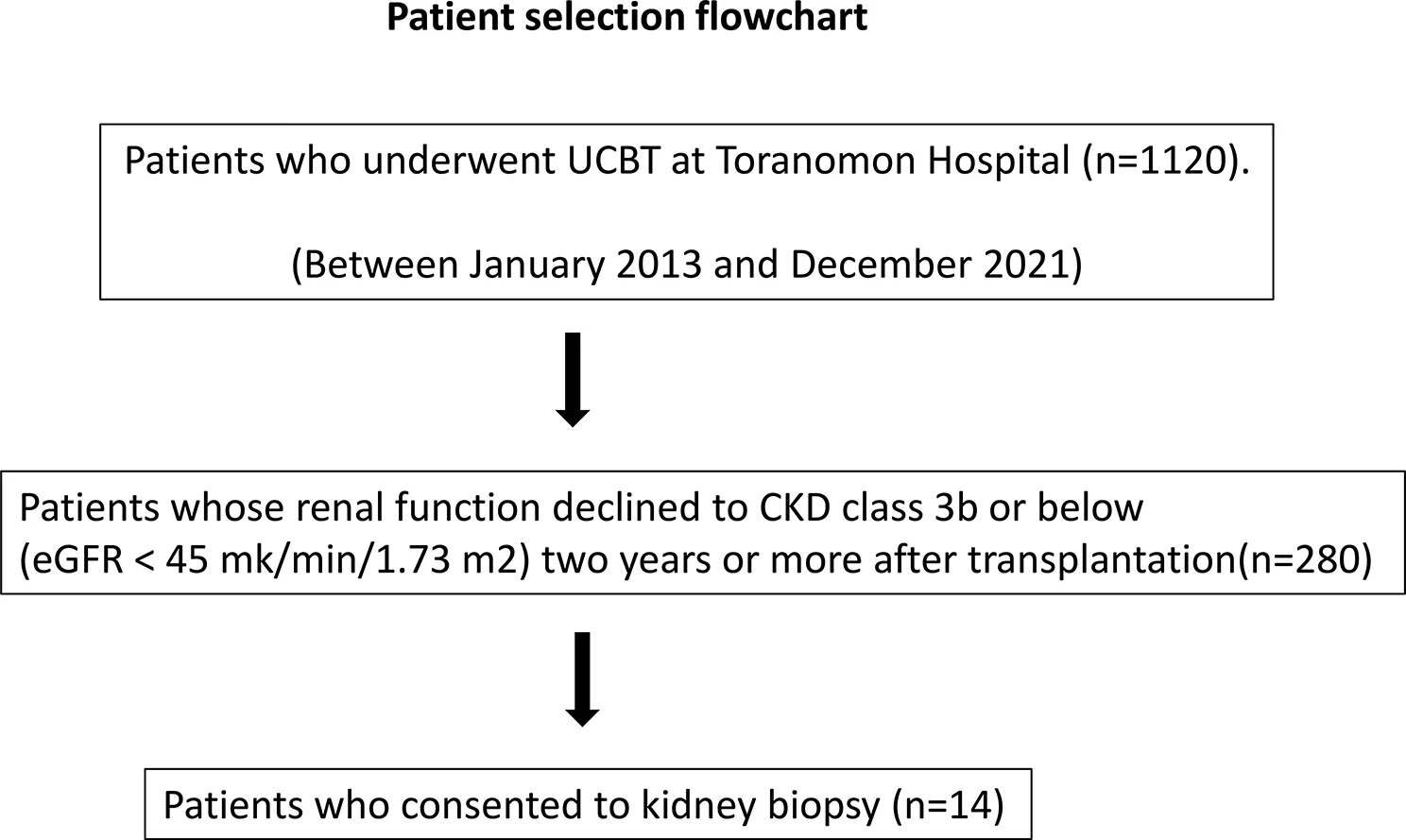
The patient selection flow chart is shown

The patient selection flow chart is shown below. From January 2013 to December 2021, a total of 1,120 patients underwent UCBT at Toranomon Hospital. Of these patients, 280 experienced a decline in renal function to CKD class 3b or below (eGFR < 45 mL/min/1.73 m^2^) two years or more after transplantation. Of these, 14 patients with proteinuria but decreased renal function compared to pre-transplantation gave consent for kidney biopsy at Toranomon Hospital between January 2015 and December 2023. We reviewed kidney biopsy reports these patients at least four renal pathologists and/or nephrologists (Fig. 1).

### Information about UCBT and kidney biopsy

The following information was retrieved from electronic medical records: Patients who underwent UCBT were included in the study; sex; age at kidney biopsy; year HSCT started; year of kidney biopsy (Table 1); history of hypertension (HT) and diabetes mellitus (DM) requiring antihypertensive drugs and diabetic medications; underlying disease requiring HSCT; number of HSCTs; number of HLA mismatches; ABO incompatibility, yes (+) or no (-); conditioning regimen; graft-versus-host disease (GVHD) prophylaxis; duration of calcineurin inhibitor (CNI) administration in months; radiotherapy energy units; organ affected by acute or chronic GVHD; serum creatinine (Cr) and estimated glomerular filtration rate (eGFR) at HSCT; platelet count at kidney biopsy; time from HSCT to kidney biopsy in months; treatment after kidney biopsy; last eGFR and proteinuria values; outcome after kidney biopsy (Table 2); serum creatinine, GFR and proteinuria immediately before kidney biopsy; reason for kidney biopsy; diagnosis of NS or CKD; and CKD stage (1, 2, 3a, 3b, 4, or 5). Kidney biopsy findings were as follows: major glomerular disease, additional glomerular disease, global sclerosis ratio (global glomerular sclerosis/total glomeruli), small renal artery lesion, interstitial fibrosis and tubular atrophy (IFTA), immunofluorescent microscopy (IF) findings in glomeruli, and complement C4d stain positivity (glomeruli and peritubular capillary [PTC]) (Table 1).

**Table 1:** Patient characteristics at the time of kidney biopsy

HSCT type	Case	Age, yr	Sex	Serum Creatinine at biopsy, mg/dL	eGFR at biopsy, mL/min/1.73 m2	Proteinuria at biopsy, g/day	Biopsy Indication	CKD stage	Major glomerular disease(LM+EM)	aditional glomerular disease	global sclerosis	small renal artery lesion	IFTA	IF(on glomeruli)	IF(C4d)
UCBT(allo)	1	27	F	2	26.2	0.23	CKD	4	GMA		18/28	VMA	vascular type fibrosis (25–50)	IgM, C3	Glo and PTC
	2	30	M	4.1	18.7	1.24		4	GMA	foamy material in the glomerular capillaries	1/24	VMA	extensive fibrosis (>50)	IgM	Glo and PTC
	3	38	M	2.54	24.3	0.55		4	GBM with marked GBM duplication		5/38	VMA	extensive fibrosis (>50)	negative	PTC
	4	40	F	2.61	16.4	1.87		4	GMA	FSGS and hyalinosis	1/6	VMA	extensive fibrosis (>50)	IgA,IgA1(mild)	Glo and PTC
	5	41	M	1.74	35.1	0.58		3b	GMA with balloon shaped dilation of capillary lumen		6/18	VMA	vascular type fibrosis (25–50)	IgM(mild)	Glo and PTC
	6	42	M	1.88	33.3	1.7		3b	GMA	diabetic nephropathy	19/38	arteriolar hyalinosis	extensive fibrosis (>50)	IgG(linear)	Glo and PTC
	7	42	F	1.91	24.2	1.65		4	GMA		6/22	VMA with hyalinosis	extensive fibrosis (>50)	negative	Glo and PTC
	8	43	M	1.49	42.6	0.32		3b	mild GMA	MN(stage 4)	76/95	VMA	vascular type fibrosis (25–50)	IgG and IgG1	Glo
	9	44	F	2.4	18.3	0.8		4	GMA		1/34	intact	extensive fibrosis (>50)	negative	n.d
	10	48	F	1.93	28.8	0.41		4	GMA		28/34	VMA with hyalinosis	vascular type fibrosis (<25)	IgM(mild)	Glo
	11	48	M	3.22	17.8	0.3		4	GMA	FSGS	6/17	intact	extensive fibrosis (>50)	IgM(mild)	PTC
	12	53	M	1.61	36.9	2.21		3b	GMA		29/47	VMA	vascular type fibrosis (25–50)	negative	Glo and PTC
	13	63	M	4.9	10.38	0.2		5	GMA		2/22	VMA with vacuolization	extensive fibrosis (>50)	IgG(partial)	n.d
	14	63	M	1.93	28.8	0.17		4	Ischmeic change		26/58	VMA	extensive fibrosis (>50)	IgM(mild)	Glo and PTC

**Table 2:** Information related to UCBT, kidney biopsy, and post-transplantation outcome

HSCT type	Case	Start year of HSCT	Yesr of kidney biopdy	HT	DM	Underlying disease for HSCT	Number of HSCT	HLA mismatch	ABO incompatible	Conditioning regimen	GVHD prophylaxis	duration of CNI administration, month	radiation, Gy	Acute GVHD (I:Intestinal tract, L:Liver, S:Skin)	Chronic GVHD (A:ascites, C:Cornea, E:Effusion, I:Intestinal tract, L:Liver, S:Skin)	Serum Cre/eGFR at HSCT, mg/dL, mL/min/1.73 m2	PLT at biopsy, 10 × 3 /μL	time from HSCT to kidney biopsy, month	Therapy after biopsy	Last eGFR, mL/min/1.73 m2	last Proteinuria g/day	Progress after kidney biopsy
UCBT(allo)	1	2011	2015	+	+	MS	1	2	+	Ara-C＋Flu＋Bu＋L-PAM	TAC+MTX	1	30	I, S	E	0.5/110.4	163	15	－	39.6	0.33	HD after 90 months
	2	2019, 2021	2022	－	－	ALL	2	1	+	Flu+L-PAM	TAC＋MMF	6	12	S	E	0.61/129.4	250	6	－	26.2	2.4	Death after 1 month
	3	2018, 2018	2020	－	－	ALL	2	1	+	VP-16+CY	TAC＋MMF	4	12	S	I	1.1/44.8	74	16	ARB	33.4	0.27	14 months alive
	4	2004	2020	+	－	HL	1	1	+	Flu+L-PAM	TAC	4	2	S	I	0.64/111.3	248	204	－	14.2	4.04	14 months alive
	5	2017, 2017	2022	+	－	AML	2	3	+	Flu+L-PAM	TAC＋MMF	5	－	S	S	0.6/116.9	200	60	－	30.4	0.39	12 months alive
	6	2020, 2021	2023	+	+	MDS	2	3	+	Ara-C+Flu+L-PAM	TAC	14	8	－	E	0.45/162.4	125	28	－	34.4	1.26	HD after 4 months, Death after 1 month
	7	2011	2018	+	－	ALL	1	4	−	Flu+Bu+L-PAM	TAC＋MMF	5	12	I, S	－	0.5/101.4	126	82	ARB	32.9	0.18	50 months alive
	8	2015, 2018	2022	－	－	CML	2	3	+	Ara-C+Flu+Bu+L-PAM	TAC	6	－	I	I	0.56/131.9	123	89	SGLT2-I	41.7	0.91	15 months alive
	9	2006	2008	－	－	AML	1	3	−	Ara-C+Flu+Bu+L-PAM	TAC	2	12	－	A	0.49/147.9	450	16	－	8.9	1.35	Death after 4 months
	10	2015	2023	+	－	CML	1	3	−	Ara-C+Flu+Bu+L-PAM	TAC	18	－	－	C	0.39/139.3	265	93	SGLT2-I	29	1.12	8 months alive
	11	2011	2021	+	－	AML	1	2	+	Ara-C+Flu+Bu+L-PAM	TAC	22	－	－	I, S	0.56/121.9	228	26	－	7.8	5.21	HD after 29 months
	12	2016	2022	－	－	AML	1	1	−	Ara-C＋Flu＋Bu＋L-PAM	TAC	4	－	－	S	0.75/88	520	60	ARB	29.6	1.26	20 months alive
	13	2002	2004	－	+	MDS	1	0	+	Ara-C+Flu+Bu+L-PAM	CyA	24	4	S	C, I, S	0.9/66.3	110	51	－	36.8	0.16	Death after 76 months
	14	2021	2023	－	－	MDS	1	2	−	Ara-C+Flu+Bu+L-PAM	TAC＋MMF	4	－	I	A, E	0.88/68.6	99	25	－	35.2	0.19	8 months alive

### Histopathological diagnosis

In accordance with a consensus decision made by our nephrology department, all kidney tissue specimens were obtained by percutaneous needle biopsies. Biopsy specimens were processed and stained using standard procedures in Japan [5]. All specimens were examined by light microscopy (LM), immunofluorescent study (IF) and electron microscopy (EM). IF included staining for immunoglobulin (Ig)G, IgG subtypes (IgG1, IgG2, IgG3, and IgG4), IgA, IgM, and complement C3, C1q, and C4d.

### Transplantation methods

At our hospital, HSCT was initially performed by UCBT, BMT, and PBSCT. In this paper, however, we focused on evaluating renal disease in UCBT only.

### HSCT-thrombotic microangiopathy

HSCT-thrombotic microangiopathy (TMA), referred to here as transplant-associated TMA (TA-TMA), is a serious complication that can occur after HSCT and manifests as hemolytic anemia, thrombocytopenia, decreased renal function, a rapid rise in blood pressure, and a variety of thrombotic symptoms. Infection and CNI administration are known to be causative factors. In the majority of cases, TA-TMA occurs within the first 100 days (median, 32 days) of transplantation; however, in our 14 cases, more than two years had passed. Furthermore, the patients had no clinical findings of TA-TMA at the time of kidney biopsy, and no thrombus formation was seen in the renal biopsy tissue. Even the patients with thrombocytopenia (see Table 2) had no clinical findings that would lead to a clinical diagnosis of TA-TMA.

### Naming of thrombotic microangiopathy lesions in HSCT

Thrombotic microangiopathy (TMA) is diagnosed if kidney biopsy shows mesangiolysis, duplication of the glomerular basement membrane (GBM), and subendothelial space expansion, even without thrombosis [2, 3]. However, some researchers have suggested that although TMA is an appropriate diagnosis for lesions with thrombi, that is not the case if there is no or only poor thrombosis formation. We used the term “glomerular microangiopathy (GMA)” descriptively to represent microangiopathic changes without thrombosis, although this terminology is not yet universally established. Similar terminology has been used in previous reports describing endothelial injury without overt thrombosis [6–8].

In the 14 patients in our study, thrombosis was assessed by phosphotungstic acid hematoxylin stain, an indicator of fibrin thrombosis, and CD61 stain, an indicator of platelet thrombosis. LM showed no significant staining in any case. Therefore, in this article we use the diagnostic name GMA rather than TMA.

In the 14 patients in this study, GMA was diagnosed when—in addition to the disappearance or reduction of mesangial cell nuclei and the loss of mesangial matrix structure—biopsy specimens showed swelling and hyperplasia of endothelial cells, edematous contents occupying the glomerular capillary lumen, GBM duplication (shown by periodic acid-methenamine silver staining), and no thrombosis formation. In addition, GMA was confirmed by EM as widening of the subendothelial space because of electron-lucent material. We noticed that the patients in this study had vascular lesions characterized by severe vessel wall thickening due to tunica media smooth muscle cell proliferation and frequent intraluminal narrowing of the small renal arteries. Therefore, to distinguish the vascular lesions from conventional atherosclerosis we defined the clinical picture as vascular microangiopathy (VMA).

The degree of interstitial fibrosis and tubular atrophy (IFTA) in the cortical area was classified as grade 1 (<25%; *n* = 2), grade 2 (25%-50%; *n* = 3), or grade 3 (>50%; *n* = 9). We also noted that the characteristic extensive tubular fibrosis of IFTA (>50%) with tubular basement membrane (TBM) duplication existed unrelated to glomerular sclerosis or arteriolar stenotic lesions.

### Statistical analysis

Data are presented as the number (percent) for categorical variables and the mean ± standard deviation for continuous variables with a normal distribution or median (interquartile range, IQR) for those with a skewed distribution. Analyses were performed with Stata/IC software ver. 12.1 (StataCorp LP).

## Results

### Patient characteristics

Kidney biopsy findings and clinical information on the period from transplantation to kidney biopsy and information on outcomes after kidney biopsy are given in Tables 1 and 2.

### Comparison of clinical findings before kidney biopsy

None of the patients fulfilled established diagnostic criteria for transplant-associated thrombotic microangiopathy (TA-TMA). Specifically, no patients showed evidence of microangiopathic hemolytic anemia, including the absence of schistocytes and elevated lactate dehydrogenase levels. Although transfusion dependency was observed in some patients due to their underlying hematologic diseases, persistent transfusion dependency after stabilization of the primary disease was not present. In addition, progressive thrombocytopenia was not observed. Furthermore, severe hypertension and other acute systemic features of TMA were absent at the time of kidney biopsy.

In the 14 patients who underwent UCBT, the median urinary protein level at the time of kidney biopsy was 0.57 g/day (IQR, 0.27–1.67 g/day); median serum creatinine, 1.97 mg/dL (IQR, 1.81–2.6 mg/dL); and median eGFR, 25.3 mL/min/1.73 m^2^ (IQR, 18.1–34.2 mL/min/1.73 m^2^) (Table 1).

Five patients (35.7%) underwent two transplants. HLA-incompatible transplants were observed in 13 patients (92.9%). ABO-incompatible transplants were observed in 9 patients (64.2%) (Table 2).

### Kidney pathology

GMA lesions were found in 13 of the 14 patients and were identified as major glomerular lesions by LM.

**Fig. 2:**
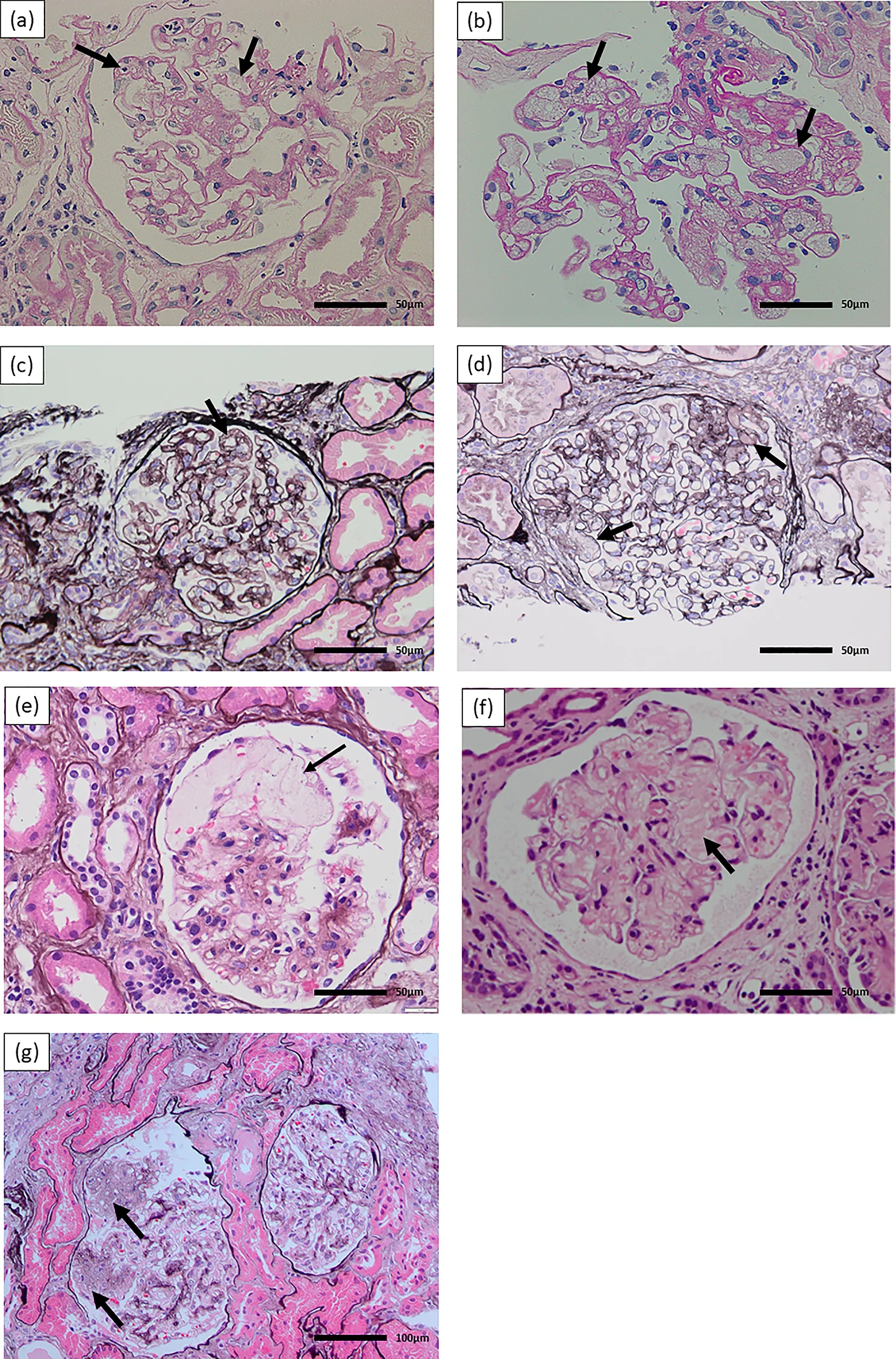

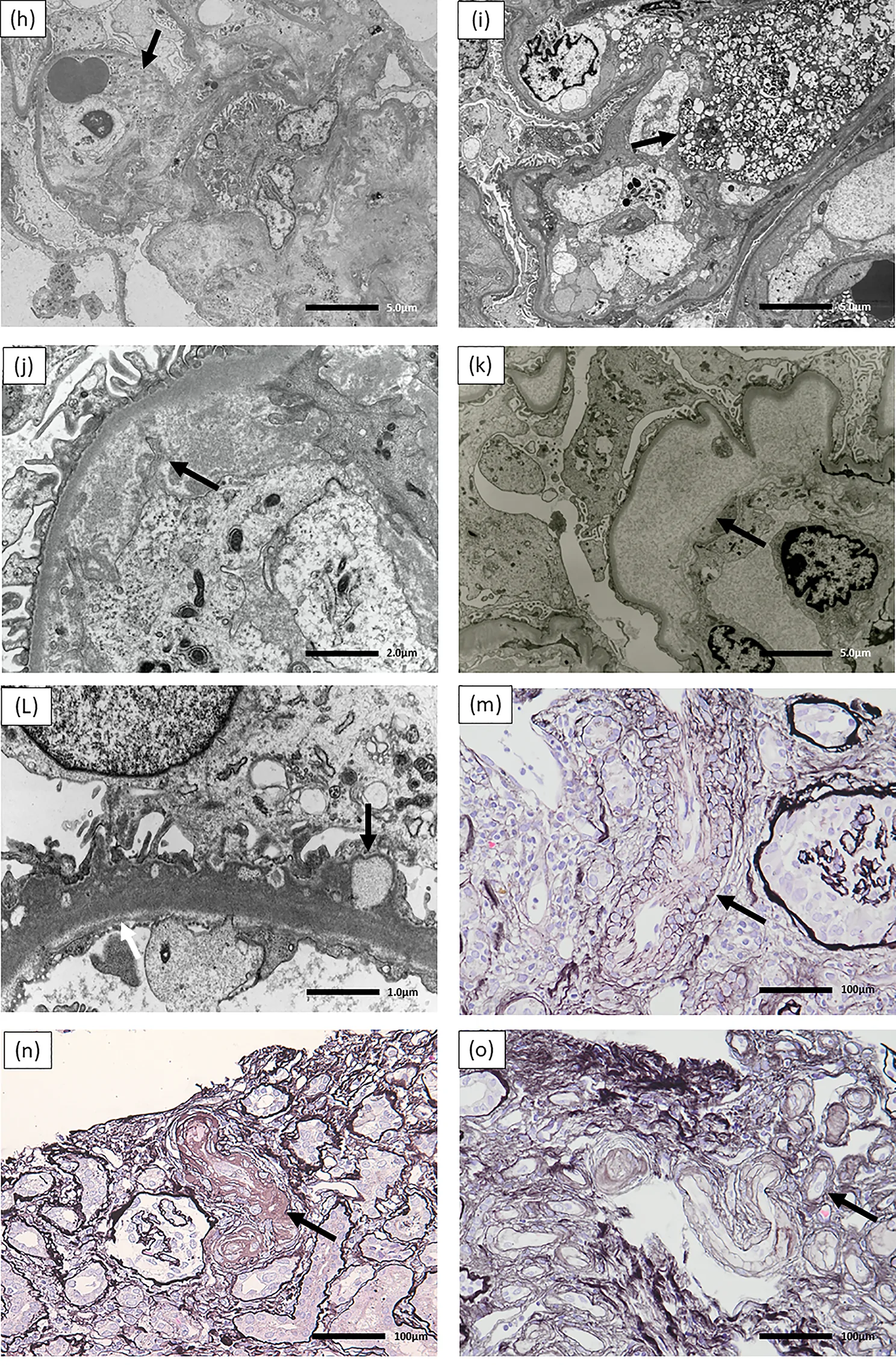

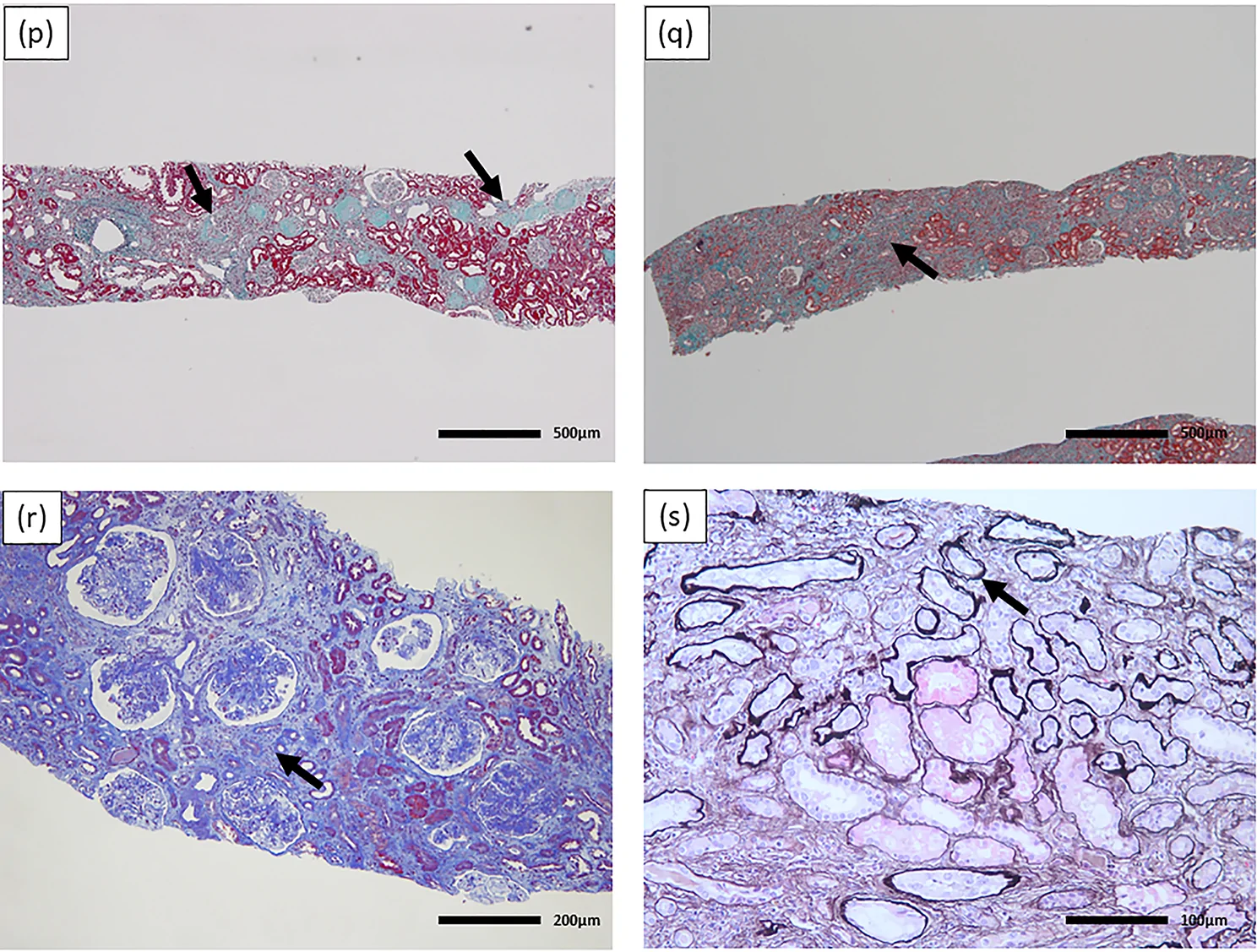
Kidney biopsy of patients with kidney disease after unrelated cord blood transplant. 2**a**-2 **h**: glomerular microangiopathy (GMA) lesions visualized by light microscopy (LM). 2**a**: complete disappearance of mesangial cell nuclei and mesangial matrix structure (arrow) in the glomerular capillary (case 1). (original magnification × 400). 2**b**: inclusion of foamy material (arrow) in the glomerular capillary (case 2). (original magnification × 400). 2**c**: marked glomerular basement membrane (GBM) duplication associated with newly formed basement membrane (arrow; case 3). (original magnification × 400). 2**d**: focal segmental sclerosis with hyalinosis (arrow) in the glomeruli (case 4). (original magnification × 400). 2**e**: balloon-like dilated capillary lumen with equally edematous material (arrow; case 5). (original magnification × 400). 2**f**: complete replacement of the capillary lumen by rough edematous material (arrow; case 9). (original magnification × 400). 2 **g**: nodular-like lesion (arrow; case 10). (original magnification × 200). 2 **h**-2 **L**: GMA lesions visualized by electron microscopy. 2 **h**: edematous widening of the subendothelial space by rough effusion material (arrow; case 1). (original magnification × 4000). 2**i**: inclusion of foamy material (arrow) in the glomerular capillaries (case 2). (original magnification × 4000). 2**j**: GBM duplication associated with newly formed basement membrane (arrow; case 3). (original magnification × 10000). 2**k**: edematous opening of the subendothelial space by equally electron-lucent material (arrow; case 9). (original magnification × 4000). 2 **L**: one patient showed old (stage 3–4) membranous nephropathy (MN) with lucent subendothelial deposits (large arrow) and a mild GMA lesion with subendothelial edema (white arrow; case 7). (original magnification × 20000). 2 **m**-2**o**: vascular microangiopathy (VMA) lesions visualized by LM. 2 **m**: pronounced vessel wall thickening due to tunica media smooth muscle cell proliferation (arrow; case 3). (original magnification × 200). 2**n**: VMA lesion with hyalinosis (arrow; case 7). (original magnification × 200). 2**o**: arteriolar proliferation (arrow; case 4). (original magnification × 200). 2**p**-2**s**: interstitial fibrosis and tubular atrophy (IFTA) lesion visualized by LM. 2**p**: linear IFTA (<25 and 25%-50%; arrow) associated with ischemic arteriolar occlusion (case 1). (original magnification × 40). 2**q**: extensive fibrosis of IFTA (>50%) (grade 3; green area indicated by arrow) in the cortical region was interstitial fibrosis unrelated to global glomerulosclerosis (case 3). (original magnification × 40). 2 **r**: extensive fibrosis (blue area indicated by arrow) similar to Fig. 1q(case 9). (original magnification × 100). 2**s**: extensive fibrosis accompanied by tubular basement membrane duplication (arrow) (case 3). (original magnification × 200)

LM identified diverse pathological features of GMA, i.e., the complete disappearance of mesangial cell nuclei and mesangial matrix structure (case 1, Fig. 2a), inclusion of foamy material in the glomerular capillaries (case 2, Fig. 2b), marked GBM duplication associated with newly formed basement membrane (case 3, Fig. 2c), focal segmental sclerosis with hyalinosis (case 4, Fig. 2d), balloon-like dilated capillary lumen with equally edematous material (case5, Fig. 2e), complete replacement of the capillary lumen by rough edematous material (case 9, Fig. 2f), and a nodular-like lesion (case 10. Fig. 2g).

EM showed diverse structures, including edematous widening of the subendothelial space by rough effusion material (case 1, Fig. 2h), inclusion of foamy material in the glomerular capillaries (case 2, Fig. 2i), GBM duplication associated with newly formed basement membrane (case 3, Fig. 2j), and edematous opening of the subendothelial space by electron-lucent material (case 9, Fig. 2k). One patient (case 7) showed old (stage 3–4) membranous nephropathy (MN) with lucent subendothelial deposits (case 7, Fig. 2L), as well as mild GMA with subendothelial edema (case 7, Fig. 2L).

Eleven patients had small artery lesions, which we termed VMAs. The lesions included severe vessel wall thickening due to tunica media smooth muscle cell proliferation (case 3, Fig. 2m), in one case also with hyalinosis (case 7, Fig. 2n), and arteriolar proliferation (case 4) (Fig. 2o).

Linear fibrosis of IFTA (<25 and 25%-50%) associated with ischemic arteriolar occlusion was observed in 5 cases (case 1, Fig. 2p). Extensive fibrosis of IFTA (>50%; grade 3) unrelated to global glomerulosclerosis in the cortical region was seen in 9 cases (cases 3 and 9, Fig. 2q+2 r), and was accompanied by TBM duplication (case 3, Fig. 2s).

### Significance of C4d

C4d was stained in 13 cases. Eleven cases were positive for glomeruli, and nine cases were positive for PTC (Table 1).

### Impact of radiation on renal tissue

Eight of the 14 patients received radiation. There was no evidence of a difference in renal tissue between those who received radiation and those who did not (Table 2).

### Involvement of CNIs in patients’ diagnosis

All 14 patients included in this study received CNIs for acute and chronic GVHD immediately after transplantation; the doses were adjusted accordingly. However, the kidney biopsies evaluated in this study were performed more than two years after transplantation, and at that time, none of the patients were taking CNIs and none had clinical features of TA-TMA.

### Outcomes after kidney biopsy

Of the 14 patients, 3 patients underwent hemodialysis after 1 to 3 months, and 3 patients died (after 1 month, 4 months, and 6.3 years) because of worsening of the underlying disease. The other 8 patients had stable renal function during 8–50 months follow-up (Table 2).

## Discussion

Most cases of kidney disease that occur after HSCT and are diagnosed by kidney biopsy are either MN or TMA-like lesions. Until now, findings in patients with kidney disease after HSCT by UCBT, BMT, and PBSCT have been reported together, and the characteristics of renal lesions specific to UCBT have not been clarified.

Mii et al. reported kidney disease in one BMT and three UCBT patients with severe endothelial injury, globally enlarged subendothelial space, double GBM contour, and mesangiolysis, with diffuse glomerular C4d deposition and subendothelial widening on EM. They termed this pathological TMA after HSCT, though thrombosis details were not described [2]. Roy et al. reviewed HSCT kidney disease subdivided as CKD, NS, and AKI; five CKD cases (PBSCT *n* = 3, UCBT *n* = 2) were diagnosed with TMA showing mesangiolysis, GBM duplication, and subendothelial expansion [3]. MN was reported in three NS cases (PBHCT *n* = 2, BMT *n* = 1) complicated by TMA with fibrin thrombosis, FGS tip variant, or FGS not otherwise specified. AKI occurred in three PBHCT and one UCBT case: one from BK virus infection with TMA and thrombosis, three from acute tubular injury [3].

Girsberger et al. described TMA, CNI-associated arteriolopathy, and MN after HSCT but did not link them to specific HSCT procedures. In their article, they emphasized differences between autopsy and biopsy findings and wrote that biopsy findings often reflect chronic, progressive disease, with mild cases and usually no thrombus formation, whereas autopsy findings often reflect acute, progressive disease, with fungal or bacterial infections, tumor infiltration by lymphoproliferative disorders, and severe TMA with thrombus formation within glomeruli and arterioles [9].

Classical thrombotic thrombocytopenic purpura presents with thrombopenia, hemolytic anemia, renal failure, fever, and encephalopathy, with TMA diagnosed when thrombosis is observed. Such patients are often too ill for biopsy, so pathology is usually confirmed postmortem. Recently, biopsy findings of mesangial injury, endothelial proliferation, or subendothelial edema have been labeled TMA even without thrombosis or thrombocytopenia. In our patients, clear thrombosis was absent; therefore, we used the term GMA instead of TMA.

The pathogenesis of GMA remains unclear. Sakai et al. suggested that mesangial cells counter glomerular capillary expansion, preventing GBM rupture. Damage or loss of mesangial cells reduces this traction, allowing outward expansion, plasma flow toward the endothelium, endothelial hyperplasia, swelling, and subendothelial edema [10]. All 14 cases showed acute or chronic GVHD. Furthermore, C4d deposition, which is considered an indicator of GVHD, was observed in 12 of the 14 cases (Table 1). We hypothesize that GMA may originate from mesangial cell injury and subsequently progress to endothelial damage; however, while C4d may reflect endothelial injury and complement activation, its specificity for GVHD-related mechanisms has not been established.

Vascular and interstitial lesions also occur after HSCT. Girsberger et al. described CNI-related lesions with arteriolar hyalinosis and striated tubulointerstitial fibrosis [9]. We found similar lesions (termed vascular-type fibrosis) in seven cases, but also identified unique features not previously emphasized: vascular microangiopathy (VMA) with severe vessel wall thickening from smooth muscle proliferation and frequent luminal narrowing, and extensive IFTA (>50%) with tubular basement membrane duplication. These findings, along with positive C4d staining in glomeruli and peritubular capillaries, suggest UCBT-specific vascular injury distinct from classic CNI nephropathy.

Radiation has been reported to be associated with renal injury after hematopoietic stem cell transplantation (HSCT) [11]. However, in this cohort, 8 of 14 patients had received radiation exposure, but similar GMA was observed in unexposed patients, indicating that radiation may contribute to renal injury but is not the only cause.

The C4d staining findings in renal tissue from biopsy and autopsy specimens reported by Laskin et al. are useful for interpreting the significance of C4d staining [12]. In cases with proven TA-TMA, C4d staining was primarily observed in the arterioles and also to a lesser degree in the peritubular capillaries and tubular basement membranes. In the control group, no staining was observed in these areas, so the positive staining was reported as being specific to TA-TMA. On the other hand, glomerular deposition of C4d was positive also in the control group, suggesting that this finding is not specific to TA-TMA. TA-TMA is characterized by endothelial dysfunction, and C4d deposition is thought to result from the associated activation of the classical complement pathway. However, an association of endothelial dysfunction with GVHD has not been conclusively proven. In our cases, C4d staining was frequently observed, primarily in arterioles, suggesting that endothelial cell damage in arterioles is related to the onset of renal lesions.

Our study population comprised patients with chronic-phase renal damage that manifested more than two years after transplantation. In contrast, previously reported cases of HSCT-TMA with thrombus formation were characterized by acute multi-organ disease that developed 20 to 100 days after transplantation, as described in the aforementioned report by Girsberger et al. [9]. TA-TMA presents with thrombocytopenia and hemolytic anemia within a short period of time and with rapid decline in renal function requiring dialysis and concurrent malignant hypertension. It is associated with infections such as cytomegalovirus and human herpes virus-6, acute GVHD, and acute CNI toxicity. Because of severe thrombocytopenia, kidney biopsy is not possible, and histological findings are primarily based on autopsy information [13]. However, this acute situation differs from the chronic-phase condition seen in our study cohort.

The patients with nephropathy in UCBT included in our study had little proteinuria more than two years after transplantation and underwent renal biopsies because of decreased renal function; they also did not have any complications of MN. On the other hand, another study found that some patients who underwent HSCT with BMT were found to have MN, which developed as nephrotic syndrome characterized by severe proteinuria more than two years after transplantation [14]. Sethi et al. identified a novel protein, protocadherin FAT1, in nine PLA2R-negative post-transplant MN patients and reported the patients’ diagnosis as transplant-specific MN.

In summary, UCBT-associated kidney disease typically manifests as CKD with mild proteinuria but decreased renal function. Pathologically, GMA, VMA, and widespread IFTA (>50%) are observed. However, definite MN has not been observed in UCBT. Previous reports indicate that BMT and PBSCT may be involved in the development of MN.

This study is subject to selection bias, as kidney biopsy was performed only in patients with clinically significant renal dysfunction. Therefore, the findings may not represent the full spectrum of renal injury after UCBT. The causal relationship between these pathological findings and GVHD remains speculative. Other factors, including calcineurin inhibitor toxicity, prior radiation exposure, and chronic endothelial injury, may also contribute to the observed lesions. Therefore, further studies with larger cohorts are required to validate these findings and clarify the underlying mechanisms.

## Data Availability

The datasets used and/or analyzed during the current study are available from the corresponding author on reasonable request.

## Abbreviations

AA: Aplastic anemia
AA: Aplastic anemia
AKI: Acute kidney injury
ALL: Acute lymphocytic leukemia
Allo: Allogeneic
ALZ: Alemtuzumab
AML: Acute myeloid leukemia
Ara-C: Cytarabine
ARB: Angiotensin receptor blocker
ARNI: Angiotensin receptor-neprilysin inhibitor
ATL: Adult T-cell leukemia-lymphoma
Auto: Autologous
BMT: Bone marrow transplantation
BPDCN: Blastic plasmacytoid dendritic cell neoplasm
Bu: Busulfan
CBT: Cord blood transplantation
CKD: Chronic kidney disease
CML: Chronic myeloid leukemia
CNI: Calcineurin inhibitor
Cr: Creatinine
CY: Cyclophosphamide
CyA: Ciclosporin A
DLBCL: Diffuse large B-cell lymphoma
DM: Diabetes mellitus
eGFR: Estimated glomerular filtration rate
EM: Electron microscopy
Flu: Fludarabine
FSGS: Focal segmental glomerulosclerosis
GBM: Glomerular basement membrane
GMA: Glomerular microangiopathy
GVHD: Graft-versus-host disease
HD: Hemodialysis
HL: Hodgkin’s lymphoma
HLA: Human leukocyte antigen
HSCT: Hematopoietic stem cell transplantation
HT: Hypertension
IF: Immunofluorescent microscopy
IFTA: Interstitial fibrosis and tubular atrophy
ITP: Idiopathic thrombocytopenic purpura
LM: Light microscopy
L-PAM: 4-bis(2-chloroethyl)-amino-L-phenylalanine, melphalan
MCNU: Methyl 6-[3-(2-chloroethyl)-3-nitrosoureido]-6-deoxy-α-D-glucopyranoside (ranimustine)
MDS: Myelodysplastic syndrome
MMF: Mycophenolate mofetil
MN: Membranous nephropathy
MS: Myeloid sarcoma
MTX: Methotrexate
NHL: Non-Hodgkin’s lymphoma
NS: Nephrotic syndrome
PAM: Periodic acid-methenamine silver
PAS: Periodic acid-Schiff stain
PBSCT: Peripheral blood stem cell transplantation
PCHCT: Peripheral blood hematopoietic cell transplantation
PLT: Platelet
PSL: Prednisolone
PTAH: Phosphotungstic acid hematoxylin
PTC: Peritubular capillary
RTX: Rituximab
SGLT2-I: Sodium glucose cotransporter 2 inhibitor
TAC: Tacrolimus
TBM: Tubular basement membrane
IF: Interstitial fibrosis
UCBT: Unrelated cord blood transplant
VMA: Vascular microangiopathy
VP-16: Etoposide

## References

[1] Ando M. An overview of kidney disease following hematopoietic cell transplantation. Intern Med. 2018, Jun, 1;57(11):1503–08. 10.2169/internalmedicine.9838-17. 29321440 PMC6028680

[2] Mii A, Shimizu A, Kaneko T, Nakayama K, Yamaguchi H, Tsuruoka S. Renal thrombotic microangiopathy after hematopoietic stem cell transplantation: involvement of chronic graft-versus-host disease. Kidney Int Rep. 2018, Jan, 5;3(3):743–47. 10.1016/j.ekir.2017.12.013. 29854984 PMC5976851

[3] Roy G, Iordachescu I, Royal V, Lamarche C, Ahmad I, Nadeau-Fredette AC, et al. Kidney biopsy findings among allogenic hematopoietic stem cell transplant recipients with kidney injury: a case series. Kidney Med. 2023, May, 16;5(7):100674. 10.1016/j.xkme.2023.100674. 37492111 PMC10363560

[4] Hiramatsu R, Ubara Y, Sawa N, Hasegawa E, Kawada M, Imafuku A, et al. Clinicopathological analysis of allogeneic hematopoietic stem cell transplantation–related membranous glomerulonephritis. Hum Pathol. 2016, Apr;50:187–94. 10.1016/j.humpath.2015.12.005. 26997455

[5] Ubara Y, Kawaguchi T, Nagasawa T, Miura K, Katsuno T, Morikawa T, et al. Committee of practical Guide for kidney biopsy 2020. Kidney biopsy guidebook 2020 in Japan. Clin Exp Nephrol. 2021, Apr;25(4):325–64. 10.1007/s10157-020-01986-6. 33606126 PMC7966701

[6] Mizuno H, Sawa N, Watanabe S, Ikuma D, Sekine A, Kawada M, et al. The clinical and Histopathological feature of renal manifestation of TAFRO syndrome. Kidney Int Rep. 2020, May, 19;5(8):1172–79. 10.1016/j.ekir.2020.05.004. 32775816 PMC7403508

[7] Yoshimura Y, Sawa N, Matsuoka S, Ikuma D, Oba Y, Sekine A, et al. Glomerular microangiopathy with cellular Crescent-like formation and Endotheliopathy due to ramucirumab treatment for metastatic sigmoid colon cancer. Intern Med. 2022, Dec, 1;61(23):3547–52. 10.2169/internalmedicine.9185-21. 35569979 PMC9790787

[8] Pfister F, Amann K, Daniel C, Klewer M, Büttner A, Büttner-Herold M. Characteristic morphological changes in anti-VEGF therapy-induced glomerular microangiopathy. Histopathology. 2018, Dec;73(6):990–1001. 10.1111/his.13716. 30014486

[9] Girsberger M, Halter JP, Hopfer H, Dickenmann M, Menter T. Kidney Pathology after hematologic cell transplantation—A single-center observation study of Indication biopsies and autopsies. Biol Blood Marrow Transplant. 2018, Mar;24(3):571–80. 10.1016/j.bbmt.2017.11.008. 29155318

[10] Sakai T, Lemley KV, Hackenthal E, Nagata M, Nobiling R, Kriz W. Changes in glomerular structure following acute mesangial failure in the isolated perfused kidney. Kidney Int. 1992, Mar;41(3):533–41. 10.1038/ki.1992.76. 1573824

[11] Cohen EP, Pais P, Moulder JE. Chronic kidney disease after hematopoietic stem cell transplantation. Semin Nephrol. 2010, Nov;30(6):627–34. 10.1016/j.semnephrol.2010.09.010. 21146127 PMC3005300

[12] Laskin BL, Maisel J, Goebel J, Yin HJ, Luo G, Khoury JC, et al. Renal arteriolar C4d deposition: a novel characteristic of hematopoietic stem cell transplantation-associated thrombotic microangiopathy. Transplantation. 2013;96(2):217–23. 10.1097/TP.0b013e31829807aa. 23698598 PMC5654605

[13] Khosla J, Yeh AC, Spitzer TR, Dey BR. Hematopoietic stem cell transplant-associated thrombotic microangiopathy: current paradigm and novel therapies. Bone Marrow Transpl. 2018, Feb;53(2):129–37. 10.1038/bmt.2017.207. 28967899

[14] Sethi S, Madden B, Casal Moura M, Nasr SH, Klomjit N, Gross L, et al. Hematopoietic stem cell transplant-membranous nephropathy is associated with protocadherin FAT1. JASN. 2022, May;33(5):1033–44. 10.1681/ASN.2021111488. 35321939 PMC9063902

