# Correction to: Kidney biopsy analysis of 14 cases of renal disease after unrelated cord blood transplantation

**DOI:** 10.1186/s12882-026-05380-4

**Published:** 2026-09-22

**Authors:** Masato Mizuta, Naoki Sawa, Tatsuya Suwabe, Yuki Oba, Akinari Sekine, Masayuki Yamanouchi, Hiroki Mizuno, Noriko Inoue, Kiho Tanaka, Eiko Hasegawa, Atsushi Wake, Junichi Hoshino, Kei Kono, Kenichi Ohashi, Yutaka Yamaguchi, Makoto Fukuda, Motoaki Miyazono, Takehiko Wada, Yoshifumi Ubara

**Affiliations:** 1 Nephrology Center and Okinaka Memorial Institute for Medical Research, Toranomon Hospital, Tokyo, Japan; 2 Department of Hematology, Toranomon Hospital, Tokyo, Japan; 3 Department of Nephrology, Tokyo Women’s Medical, University, Tokyo, Japan; 4 Department of Pathology, Toranomon Hospital, Tokyo, Japan; 5 Department of Human Pathology, Institute of Science Tokyo, Tokyo, Japan; 6 Yamaguchi’s Pathology Laboratory, Chiba, Japan; 7 Department of Nephrology, Saga University Internal Medicine, Saga, Japan

**Correction to: BMC Nephrology (2026) 27:414**

**https://doi.org/10.1186/s12882-026-05034-5**

In Fig. 2 of this article [3 figure panels (Fig. 2h-2k, Fig. 2l-2o, Fig. 2p-2s) were missing]; the figure panels were given as Fig. 2a-2d, Fig. 2e-2g but should have appeared as Fig. 2a-2d, Fig. 2e-2g, Fig. 2h-2k, Fig. 2l-2o, Fig. 2p-2s.

**Fig. 2:**
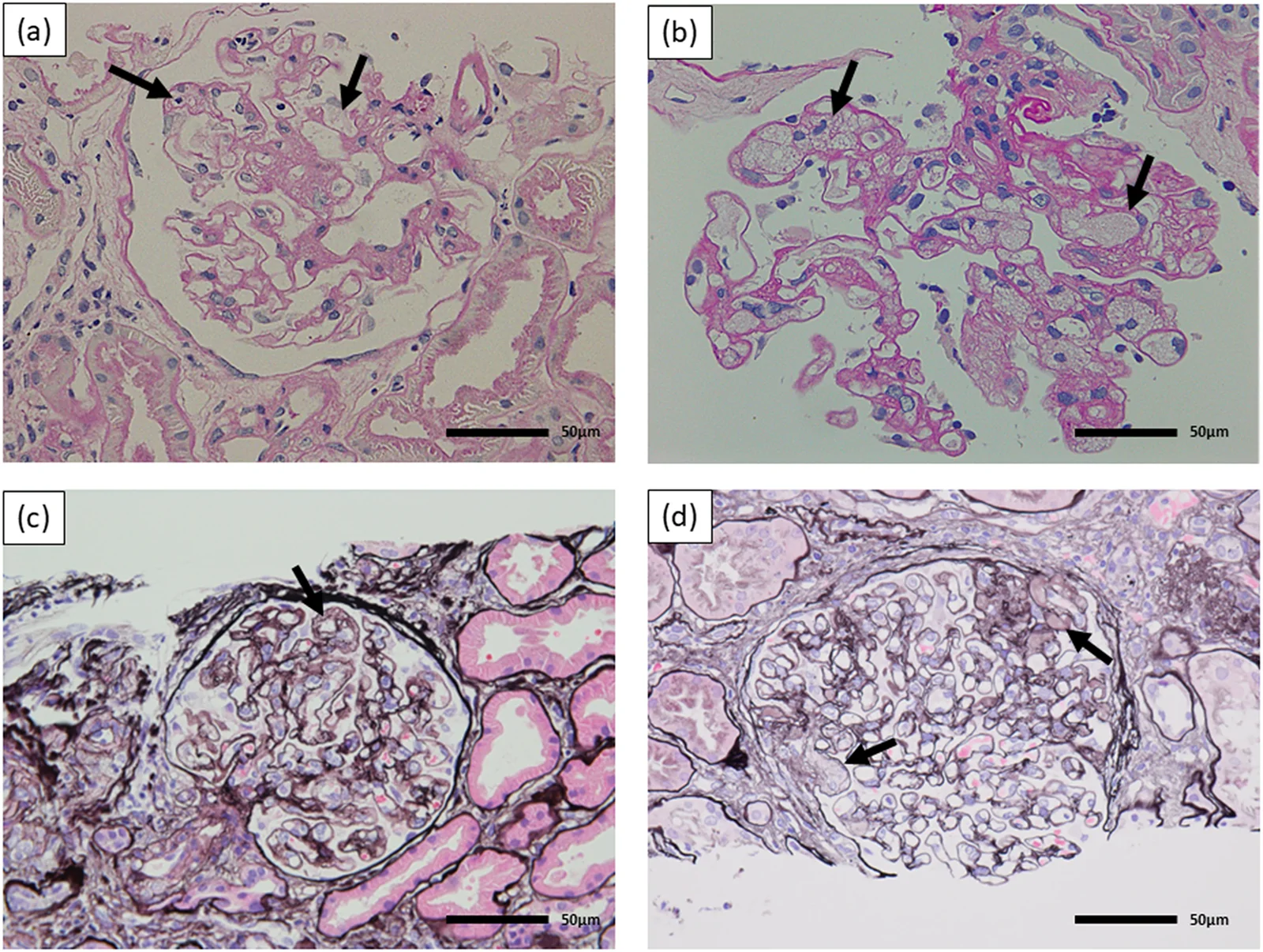

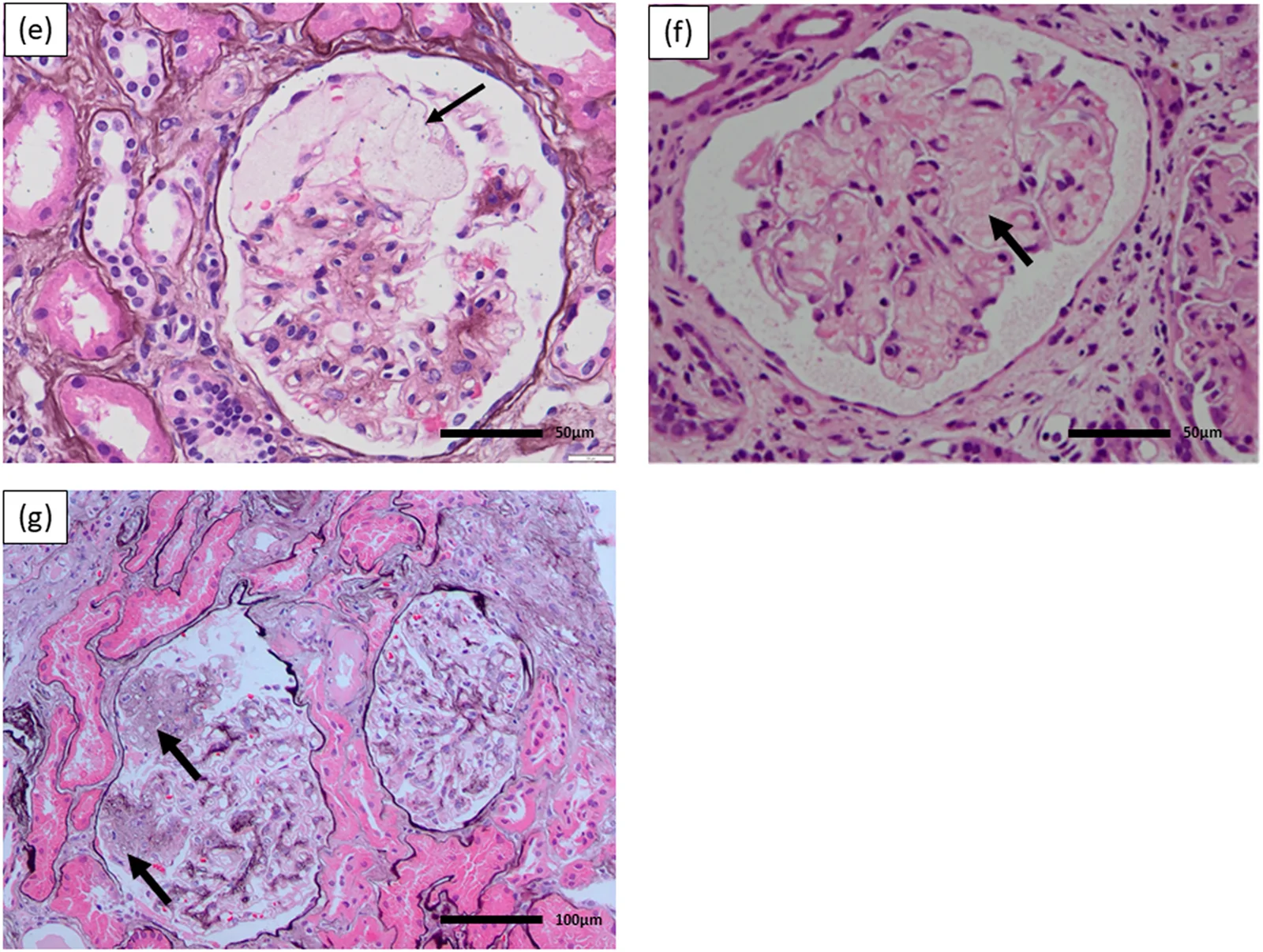

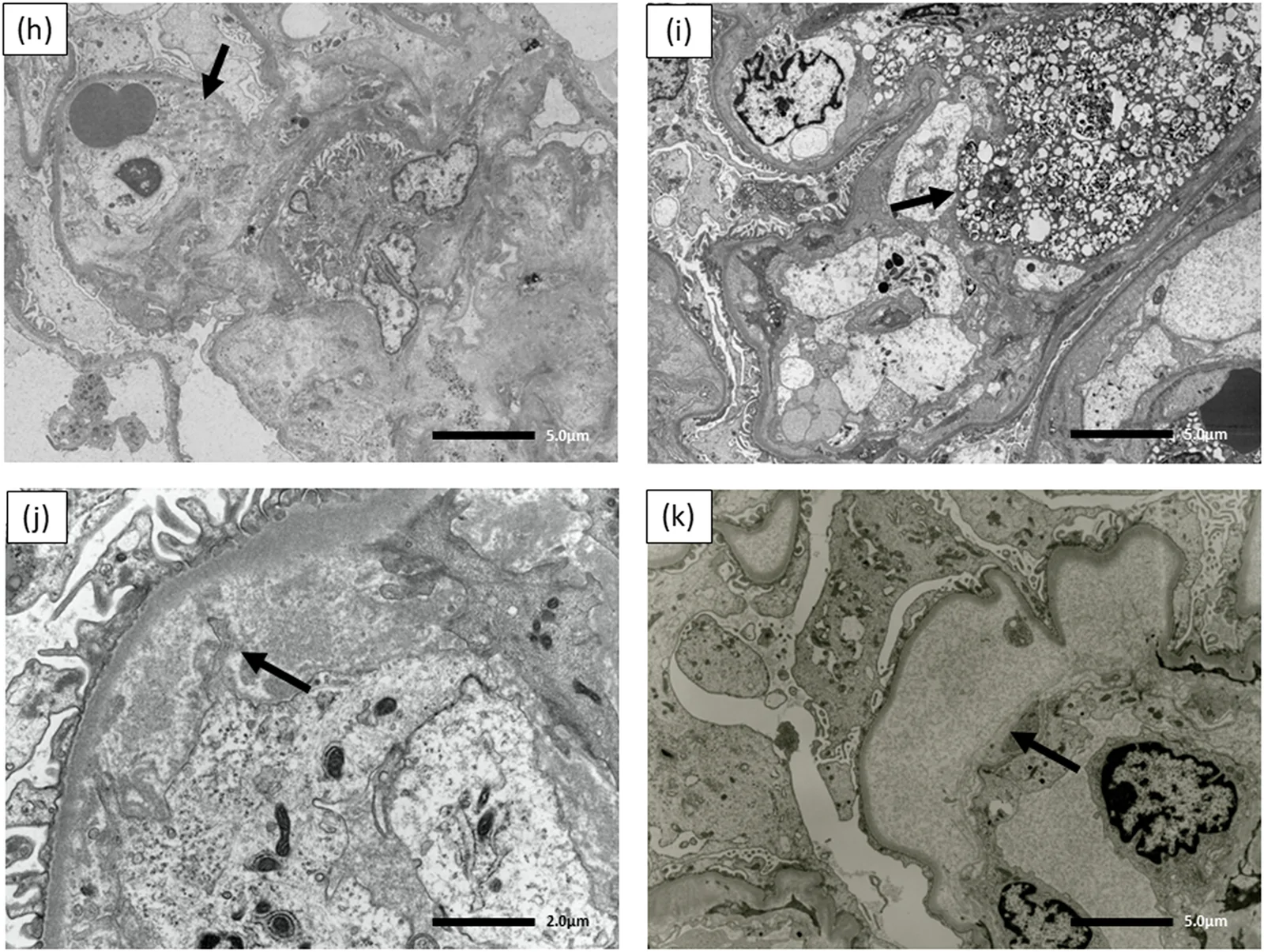

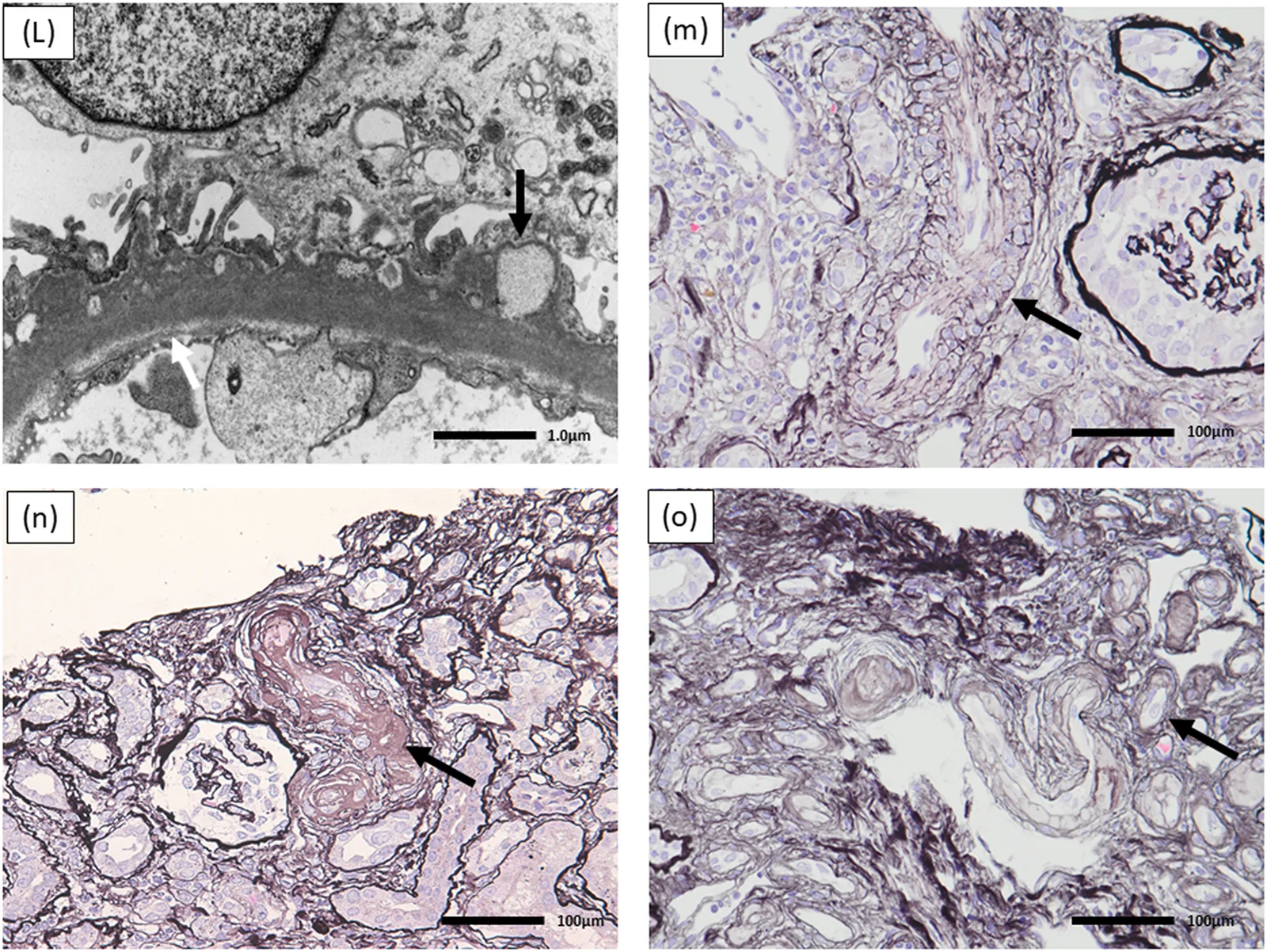

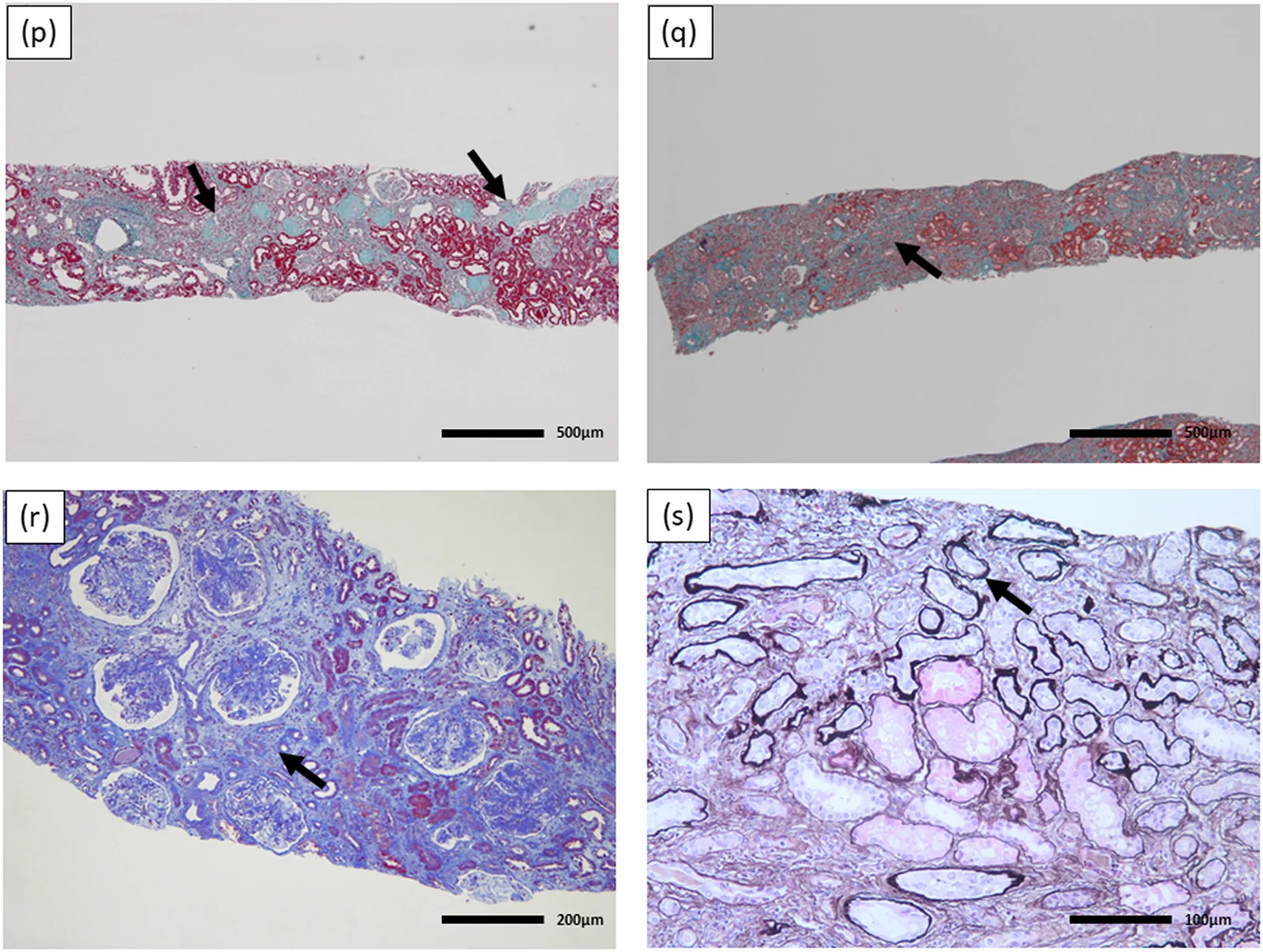
Kidney biopsy of patients with kidney disease after unrelated cord blood transplant. 2**a**-2 **h**: glomerular microangiopathy (GMA) lesions visualized by light microscopy (LM). 2**a**: complete disappearance of mesangial cell nuclei and mesangial matrix structure (arrow) in the glomerular capillary (case 1). (original magnification × 400). 2**b**: inclusion of foamy material (arrow) in the glomerular capillary (case 2). (original magnification × 400). 2**c**: marked glomerular basement membrane (GBM) duplication associated with newly formed basement membrane (arrow; case 3). (original magnification × 400). 2**d**: focal segmental sclerosis with hyalinosis (arrow) in the glomeruli (case 4). (original magnification × 400). 2**e**: balloon-like dilated capillary lumen with equally edematous material (arrow; case 5). (original magnification × 400). 2**f**: complete replacement of the capillary lumen by rough edematous material (arrow; case 9). (original magnification × 400). 2 **g**: nodular-like lesion (arrow; case 10). (original magnification × 200). 2 **h**-2 **L**: GMA lesions visualized by electron microscopy. 2 **h**: edematous widening of the subendothelial space by rough effusion material (arrow; case 1). (original magnification × 4000). 2**i**: inclusion of foamy material (arrow) in the glomerular capillaries (case 2). (original magnification × 4000). 2**j**: GBM duplication associated with newly formed basement membrane (arrow; case 3). (original magnification × 10000). 2**k**: edematous opening of the subendothelial space by equally electron-lucent material (arrow; case 9). (original magnification × 4000). 2 **L**: one patient showed old (stage 3–4) membranous nephropathy (MN) with lucent subendothelial deposits (large arrow) and a mild GMA lesion with subendothelial edema (white arrow; case 7). (original magnification × 20000). 2 **m**-2**o**: vascular microangiopathy (VMA) lesions visualized by LM. 2 **m**: pronounced vessel wall thickening due to tunica media smooth muscle cell proliferation (arrow; case 3). (original magnification × 200). 2**n**: VMA lesion with hyalinosis (arrow; case 7). (original magnification × 200). 2**o**: arteriolar proliferation (arrow; case 4). (original magnification × 200). 2**p**-2**s**: interstitial fibrosis and tubular atrophy (IFTA) lesion visualized by LM. 2**p**: linear IFTA (<25 and 25%-50%; arrow) associated with ischemic arteriolar occlusion (case 1). (original magnification × 40). 2**q**: extensive fibrosis of IFTA (>50%) (grade 3; green area indicated by arrow) in the cortical region was interstitial fibrosis unrelated to global glomerulosclerosis (case 3). (original magnification × 40). 2 **r**: extensive fibrosis (blue area indicated by arrow) similar to Fig. 1q(case 9). (original magnification × 100). 2**s**: extensive fibrosis accompanied by tubular basement membrane duplication (arrow) (case 3). (original magnification × 200)

